# Measuring Mothers’ Viewpoints of Breast Pump Usage

**DOI:** 10.3390/ijerph18083883

**Published:** 2021-04-07

**Authors:** Genevieve E. Becker

**Affiliations:** BEST Services, H91T22T Galway, Ireland; gbecker@bestservices.ie

**Keywords:** methodology, instruments, user viewpoint, systematic review, human milk expression, breast pump

## Abstract

Breastfeeding has short- and long-term positive influences on the health and wellbeing of the child. There are situations where breastfeeding does not occur and expressed or pumped mother’s milk is used. Mothers and healthcare providers report problems or negative views on using pumps in studies across the globe. This systematic review and secondary analysis of 18 random control trials related to mothers’ views of breast pumps examines the range of viewpoints gathered, the variety of measurement instruments used, how the outcomes are reported and the challenges that occur. It aims to inform critical reading of research as well as future research design. Devices which the mother views as comfortable and useful will facilitate more infants to receive human milk when direct breastfeeding does not occur, and they will have a positive influence on health and wellbeing.

## 1. Introduction

Breastfeeding has short- and long-term positive influences on the health and wellbeing of the child. It also has effects on the mother’s psychological, hormonal, and biochemical state, as well as family and community economics and the environment, which in turn influences the child’s health and wellbeing [1,2,3,4,5,6,7,8,9].

There are situations where breastfeeding does not occur due to temporary or ongoing separation of mother and child, very premature birth, illness, impairment, or mother’s choice, and expressed or pumped mother’s milk is used. This may be the child’s own mother’s milk or donor mother’s milk. Hand expression of milk is a minimal-cost method available worldwide; however, there is increasing development and marketing of a variety of types of breast pumps, with the world market projected to grow to USD 829 million by 2022 [10].

Two recent surveys report the aspects that mothers considered important in choosing and using a breast pump and highlight how the importance can relate to the reason for use and the setting. A survey of women in the Northeast USA currently or recently using a pump collected their views on the importance of a list of seven pump characteristics. Portability, ease of use, low weight, fast milk extraction, and comfortability were all rated to be important, with low noise and discreetness also important to younger mothers. Fifty-seven percent of the respondents were aged 20–34, 90% had completed college or higher and 79% were employed outside the home at the time of the survey [11]. This sample may have resulted in viewpoints predominately related to older babies and where portability and discreetness (low noise) may be more valued due to employment settings than if pumping was for an ill newborn in a neonatal unit.

The views of mothers who were users of pumps across eight European countries and the USA were gathered through open-question interviews as to what they consider are important aspects of choosing and using a breast pump [12]. This study also collected the views of health workers who advise or assist mothers who are using a pump. The three aspects of a pump most frequently mentioned by mothers and by health workers were the same, though the percentages differed: ease of use (including comments on assembly, low number of parts, directions, noise, weight, and portability)—86% mothers and 90% health workers; comfort (level of pain)—68% and 66%; and efficiency (amount of milk for time)—65% and 80%. Cost as an aspect of choosing a pump was mentioned similarly (30% by mothers and 28% by health workers), with a noticeable geographic variation in relation to the availability of a free pump from health insurers or other sources. Health workers mentioned hygiene-related aspects more frequently than mothers (45% versus 28%), and the effect of pumps on milk constituents (14% versus 0%); these differences may be related to the number of health workers in the sample who were working with vulnerable babies.

The viewpoint of the person using a device is an important component of equipment design, testing, and marketing, as well as the potential usage of the device. Similar to the development of other devices, the viewpoint of the mother is important for breast pump devices, though it does not appear to have been central to the process [11,13]. Mothers report problems or negative views on using pumps in both trials and in qualitative or observational studies across the globe and pain is frequently mentioned [11,14,15,16,17,18,19,20,21]. Discomfort and pain reduce the mother’s oxytocin reflex and thus inhibit the flow of milk [22]. Pain can affect the mother’s willingness to use the pump at a frequency needed to establish and maintain her milk supply [11,23]. The many reports of negative views raise the issue of which views of mothers are considered and how these views are obtained during design and testing phases.

Viewpoints can be measured through satisfaction ratings, ranking of attributes, basic yes/no responses, and open questions that are analyzed qualitatively, using focus groups, brainstorming, surveys, and interviews, as well as other methods. Concerns about the validity and reliability of instruments measuring the viewpoints of the user across various areas of study have raised doubt about the credibility of findings [24] and have indicated that ratings by individuals may not be reproducible if repeated with a tendency to regress to the mean [25].

A mother’s views on the interlinked experience of birth, the responsibility of becoming a mother and caring for a baby, commencing breastfeeding or milk expression/pumping, comfort with her body image, support systems, settings, cultural norms, and other aspects can be challenging for the researcher to separate out in order to focus only on the mother’s views on using a specific device or method related to expressing/pumping [13], particularly when comparing one device to another device or across studies. Negative attitudes to pumping were found more likely to be associated with unanticipated reasons for the use of a pump and where there was a perceived lack of support [19]; however, this study only explored the mothers’ experiences with the practice of pumping and not their views on the pumping devices specifically.

The Cochrane Review on methods of milk expression included published and unpublished, randomized or quasi-randomized, controlled trials in which one method or technique of milk expression was compared with another [18]. The interventions examined included devices and equipment used in expressing/pumping milk as well as any adjunct techniques used to assist the flow of milk. The trials employed a variety of means to measure mothers’ viewpoints. Further examination of these trials can assist the discussion and development of means to include the views of the users of pump devices.

This review examines what viewpoints were gathered, the variety of measurement instruments used, and how the outcomes were reported in trials related to mothers’ views of breast pump usage. It aims to inform critical reading of research as well as future research design.

## 2. Materials and Methods

For this current paper, a secondary analysis was undertaken of the 15 random control trials (RCTs) in the Cochrane Review [18] which reported maternal viewpoint data [26,27,28,29,30,31,32,33,34,35,36,37,38,39,40]. An additional search for more recent RCTs from March 2016 to December 2020 used the same search strategy of the Cochrane Review. This additional search found three newer trials [41,42,43] which met the inclusion criteria of the original review, which was published and unpublished, randomized or quasi-randomized, controlled trials that compared one method or technique of milk expression or pumping with another, or others. Further details of the methodology can be found in the original review [18].

A structured analysis was conducted of the aspects examined and the methods used to measure mothers’ viewpoints in these 18 trials. The eighteen studies were first tabulated, including the date of the study’s publication, brief description of the trial, inclusion or not of the aspects found important by pump users in previous research [11,12], and sample details including sample size, the time since birth/age of baby, health of baby, setting, and other relevant information provided by the study (Table 1). Then, the 18 studies were analyzed and tabulated with regard to the instruments used to measure the mothers’ views in each study and the ways in which the findings were reported (Table 2). Due to the multiple aspects included in the trials and lack of data, it was not possible to carry out a systematic assessment on the quality of the studies that would be specific to the methods of measurement of the viewpoint of the mothers.

The information on the trials analyzed is based on the published reports. For the Cochrane Review, further details were sought on the instruments reported as being used and, where obtained, this additional information was available for this secondary analysis.

Trials may include terms such as satisfaction, preference, view, evaluation, ranking, opinion, comfort, and perception; the term viewpoint is used generically in the text here.

## 3. Results

The trials were carried out at postnatal periods ranging from 12 h to 35 weeks with mothers of infants who were in neonatal units (10 trials), a mother and full-term infant on a postnatal ward (1 trial), and mothers with healthy infants at home (7 trials).

The trials reviewed had an assortment of foci (Table 1). Three trials examined the effect of adjunct behaviors such as methods of relaxation or massage on milk output as the primary outcome, with the mother’s views of the method also reported on; these trials were unrelated to a specific pump [28,32,35].

The other trials compared two or more pumps or variations of pumps, with the mother’s views focused on the characteristics of the devices: comparing a pump to another pump [27,29,30,36,37,38,41] and to hand expression [39,40,43], pump design features (including suction level [33,34] or breast shield type [42]), or patterns of use (simultaneous or sequential [26,31,32]).

**Table 1:** What mothers’ viewpoints were measured?

Study (First Author & Year) and Trial	Overall View	Ease of Use ~	Comfort or Pain	Effectiveness/Efficiency	Other	Sample
Boutte 1985 2 pumps compared	√	√	√ *	X	X	9 breastfeeding mothers of healthy infants, mean age 3.2 months
Feher 1989 audio tape of relaxation exercises	√	X	X	X	X	55 mothers of preterm infants expected to be in NICU for at least 10 days
Auerbach 1990 sequential versus simultaneous pumping	√	X	X	X	X	26 mothers of healthy infants 5–35 weeks in age, already using a pump or planning to use a pump in the future
Mersmann 1993 Therapeutic Touch	√	X	X	X	X	18 mothers of 21 non-nursing, hospitalized, preterm infants
Paul 1996 hand expression versus pump	√	X	X	X	X	36 mothers of infants in the neonatal unit (mean gestation age 34 weeks) who were unable to suck at the breast
Hill 1999 sequential versus simultaneous pumping	√	X	X	X	X	39 mothers of preterm (<32 weeks) and low-birthweight (<=1500 g) infant(s) in neonatal unit
Jones 2001 sequential versus simultaneous pumping and massage	√	X	√	√	X	36 mothers of preterm infant(s) in neonatal unit
Fewtrell 2001 a2 pumps compared	√	√	√	X	Choosing to keep using pump type	58 mothers of term (>37 weeks) and now over 6 weeks old
Fewtrell 2001 b2 pumps compared	√	√	√	X	X	118 mothers of preterm (35 weeks) infants in neonatal unit
Meier 2008 pump suction patterns compared	√	√	√	√	Choosing to keep pattern type (Protocol 1)	100 mothers of infants in neonatal unit who weighed <1250 g and/or were born 32 weeks’ gestation
Hopkinson 2009 2 pumps compared	√	√	√	X	Choosing to keep pump type, expected effect on milk supply over time	69 healthy mother of term infants at least 3 weeks postpartum
Meier 2012 pump suction patterns compared	X	√	√	√	X	128 breast-pump-dependent mothers of infants (< 34 weeks) who anticipate remaining in NICU for > 15 days
Flaherman 2012 Hand expression versus pump	X	X	√	X	Confidence, comfort being seen by others expressing/pumping	68 mothers of term healthy newborns on the postnatal unit who were latching or sucking poorly
Bernabe-Garcia 2012 4 pumps compared	√	√	√	X	Pump selected to continue using	32 mothers of singleton preterm (<37 weeks) infants in neonatal unit
Burton 2013 2 pumps compared	√	√	√	√		71 mothers of preterm infants (<34 weeks) in neonatal unit
Fewtrell. 2019 2 pumps compared plus control with no pump	√	√	√	X	Breastfeeding goal, continued use of the assigned pump	110 mothers of healthy exclusively breastfeeding infant 3–4 weeks old; not regular pump user
Francis 2019 hand expression compared with 3 pumps	X	X	√	X	X	46 mothers of healthy breastfeeding Infant aged 2–3 months. Some experience but not regular pump user
Sakalidis 2020 Breast shield design	X	√	√	X	X	49 mothers of healthy breastfeeding Infant aged 1–6 months
Number included this aspect	14	10	13	3	6	

~ Included any aspects of assembly, number of parts, ease of cleaning, instructions, noise, portability. * Measured both discomfort and pain as separate items.

### 3.1. What Viewpoints of Mothers Were Measured?

Mothers’ overall views were measured in 15 trials (Table 1). Views in relation to comfort and/or pain or “feel”, including flexibility on the rate and amount of suction and “pleasant to use”, were measured in 13 trials. Ease of use, including aspects of assembly, number of parts, ease of cleaning, instructions, sound/noise, location of control button, portability, or leakage, was measured in 10 trials. Mothers’ views on an aspect of effectiveness or efficiency were included in three trials.

Other outcomes measured in six trials included mothers’ views on confidence, comfort being seen by others expressing/pumping; expected effect on milk supply over time; and choice of a pump (or pumping pattern) to continue to use after the trial period. Their continuing choice was used to provide an indicator of maternal view, in addition to a measurement.

One recent trial [41] reported if the method of pumping was associated with mothers’ attaining their own goals regarding exclusive breastfeeding duration. None of the trials reported on mothers’ views about the financial cost of using a pump.

### 3.2. How Were Mothers’ Viewpoints Measured and Reported?

There was variety in how the mothers’ viewpoints were measured and included rating scales, interviews, and simple questionnaires, with some studies using a combination of instruments (Table 2). For most of the trials, the mother’s viewpoint was a secondary aspect of the trial and detailed information was not provided. Published information was found for only one trial that described the underlying construct, development, and testing of the measurement instrument or any psychometric properties related to reliability, internal consistency, and predictive validity [44]. One trial carried out across four countries reported the steps taken to validate the rating statements for an instrument designed in one culture and used in another culture and language [41].

**Table 2:** How viewpoints were measured and reported.

Study (First Author & Year)	Study Instrument	Reported
Boutte 1985	Interview on 7 aspects (with response as positive or negative)	Descriptively as percentage who “responded positively” to questions about each pump
Feher 1989	Phone interview and mailed questionnaire (open question)	Brief descriptive comment only
Auerbach 1990	Interview (open question)	Descriptively as percentage preferring a method and reasons for their preference (cross-over)
Mersmann 1993	Questionnaire (1 closed question and space for additional comments)	Descriptively as percentage giving reply of positive, negative, not sure
Paul 1996	Interview (no details available)	Descriptively number preferring each method and reasons
Hill 1999	Phone interview (open questions)	Descriptively number preferring each method and reasons
Jones 2001	1 question on effectiveness with analogue scale (0–8), and space for additional comment	Median and range, with additional comments reported descriptively (Protocol II cross-over)
Fewtrell 2001b	(Original) 5-aspect Fewtrell Scale (Likert-type 1–7)	Tabulated as number and percentage giving each rating for each aspect of the two pumps (cross-over)
Fewtrell 2001a	5-aspect Fewtrell Scale (Likert-type 1–7)	Ratings recoded into 5 catgories for analysis (rating of 5–7 condensed due to low numbers with these scores) and tabulated as percentage in each pump group giving the rating on each aspect
Meier 2008	13 to 18 questions or statements. 5-point Likert type-scale (protocol 1) and collapsed to 3-point (protocol 2) (1 = strongly disagree) and multiple-choice items.	Tabulated as mean score and descriptively. For Protocol 1, the 5-point scale was analyzed as 1–3 = disagree and 4–5 = agree (cross-over)
Hopkinson 2009	7-aspect (adapted) Fewtrell Scale (Likert-type 1–7) plus 3 aspects of views regarding continued use	Differences in mean ratings and continued use of pump were reported descriptively (cross-over)
Meier 2012	Questionnaires at three time points of 13–18 Likert-type and multiple-choice items derived from their previous studies	Statistically significant differences mentioned descriptively
Flaherman 2012	Breast Milk Expression Experience (BMEE) (1 = strongly disagree to 5 = strongly agree) measure formed from modified 14-item Breastfeeding Self-Efficacy Scale (BSES) (Likert-type 1–5, high score better) and modified Holdcroft Pain Scale (Rating 1–10)	Ratings tabulated with mean and SD for BSES and BMEE, number and percentage with a pain score >5, and differences reported descriptively
Bernabe-Garcia 2012	5-aspect Fewtrell Scale (Likert-type 1–7)	Tabulated with median rating and range for each of the four pumps for each aspect (cross-over)
Burton 2013	9-aspect (adapted) Fewtrell Scale (Likert-type 1–7)	Ratings were re-coded into 3 catgories for analysis using two methods, one of which gave greater emphasis to extreme scores and different results. Raw data (using the 7-point scale) presented in bar charts for each of the 9 aspects.
Fewtrell et al. 2019	9-aspect 10-cm visual analogue scale (VAS)	Reported as median (25th and 75th percentiles) descriptively
Francis 2019	Modified Wong–Baker FACES Pain Scale (6 points)	Percentage reporting pain score >3, descriptively (cross-over)
Sakalidis 2020	5 aspects devised for this study (5-point scale strongly agree-strongly disagree)	Descriptively as percentage giving reply of “strongly agree” (cross-over)

#### 3.2.1. Interviews and Simple Questionnaires

Interviews or simple questionnaires were used to collect data in six trials. These included mothers asked:

“to rate positively or negatively seven aspects of pump usage: pump assembly, pump operation, pump dismantling, pump cleaning, physical discomfort, pain or anxiety during pump usage, and pump usage for personal or research studies” [38].

“about use [of the method]” [28].

“which pumping method was preferred and why” [26].

“did the [method] help you?” and optionally to comment on how it helped [35].

“about their preference of the method” [40].

“about their use of the breast pump, pumping style, and pump style satisfaction, if applicable” [31].

Interview or simple questionnaire findings were reported descriptively with the number or percentage preferring a method plus the mothers’ comments on the reasons for their preference in some trials.

#### 3.2.2. Rating Scales

Six of trials used the Fewtrell scale or an adaptation to measure consumer-focused characteristics; four of these trials came from the same research team. The first trial [30] reporting use of the Fewtrell questionnaire asked mothers to rate their assigned pump using a seven-point analogue scale on five aspects. Subsequent adaptations and modifications to this scale used the same five aspects [29,37], seven aspects [36], and nine [27,41] aspects (Table 3). Not all researchers who adapted the Fewtrell scale displayed the ratings of 1–7 going in the same direction (i.e., if a rating of 1 was most favorable or if 1 was least favorable). A recent trial using the Fewtrell scale used a 10-cm visual analogue scale where mothers were asked to mark their level of agreement to questions on various aspects of the pump usage along a line from negative to positive, rather than the analogue scale used previously [41].

One study that used an adapted Fewtrell scale additionally asked mothers about their expectations regarding continued use for several months and the effect on milk supply (1 = decrease to 7 = increase), irritation of nipples (1 = irritate to 7 = cause no problems), and usage (to stop using it = 1 to happy to use it every day = 7) [36].

A rating scale was used with questionnaires devised for the specific study in four trials to elicit views on effectiveness, using one question with an analogue scale of 0 to 8 [32]; views on five aspects of comfort, fit, and ease of use with a five-point scale from strongly agree to strongly disagree [42]; and a Maternal Perceptions Questionnaire used in two trials from the same research team [33,34]. This Maternal Perceptions Questionnaire contained 13 to 18 questions or statements “derived from their previous studies”, with Likert-type five-point scales and multiple-choice items repeated over time periods to measure maternal perceptions of the efficiency, efficacy, comfort, and convenience of pump suction patterns, with a scale from 1 strongly disagree to 5 strongly agree.

One study [39] used a combination of their newly developed “Breast Milk Expression Experience” measure [44], which included questions about clarity of instructions when learning to express milk and comfort with expressing with other people present, a modified Holdcroft Pain Scale [45], and a modified Breastfeeding Self-Efficacy Scale Short Form to measure confidence [46], each with a rating scale.

A modified six-point Wong–Baker FACES Pain Rating Scale was used in one study, which requested the mother to mark the face depiction most appropriate to her level of pain after using the trial pump [43].

The findings from use of these scales were presented differently and included the percentage giving each rating on each aspect [29,30,42], the mean ratings [33,36], median and range of ratings [32,37], median with 25th and 75th percentiles and visually [41], mean and standard deviation and percentage giving a pain score >5 on a scale of 10 [39], percentage giving a pain score >3 on a scale of 6 [43], as well as descriptive reporting of selected aspects.

Seven trials [29,32,33,36,37,42,43] using rating scales were cross-over studies, with each study participant comparing all the methods being studied, though not all the studies presented pair-wise data.

## 4. Discussion

The majority of the trials reviewed (13/18) included mothers’ views on comfort and/or pain when using the method or equipment. Three studies referred to pain when asking mothers to rate each pump positively or negatively for physical discomfort, pain, or anxiety during usage [38] or to rate their level of pain using existing pain scales with 0–10 [39,43]. The other studies referred to comfort and asked mothers to numerically rate “pleasant to use” and “comfortable to use” on a scale (Fewtrell scale and adaptations), or how comfortable to use [33,34], or “felt comfortable” with a 5-point scale strongly agree-strongly disagree [42]. Surveys have found that some level of reported discomfort or pain is common with pumping and this expectation may affect what is asked and mothers’ responses to rating pain when using a pump. It was noted that other aspects, such as the noise level of the pump, may influence the mother’s overall perception of “comfort” if these other aspects are not measured separately [33]. Low awareness of the physiology of milk flow may result in mothers, and those assisting the mothers, believing that a pump with stronger suction would extract the milk faster and that discomfort or pain needed to be tolerated to achieve speed [12].

Ease of use is mentioned as an important aspect in surveys with mothers. It was measured in 10 of the 15 trials reviewed here. It was a broad category including rating overall “ease of use” on a scale as well as specific aspects such as ease of assembly, noise level, or location of the control button. The trials’ periods ranged from a single use of the pump or method to many days or weeks and also had a variety of timing postnatally and settings. It is reasonable to assume that a mother’s view on ease of use might change as she uses the pump for a longer period or depending on the situation in which it is used.

Effectiveness or efficiency of a method is also a subjective view. The reasons that mothers have for expressing their milk or using a breast pump are wide-ranging and their needs are likely to vary at different stages in the postnatal period. The setting and the timing of gathering mothers’ views—in the immediate postnatal days, when breastfeeding is established, with an infant in a neonatal unit, for use in a workplace, if the baby is also feeding at the breast or if the mother is exclusively pumping, and if the trial data are gathered contemporaneously or retrospectively—is likely to be important and worthy of consideration in examining the design and findings of trials in relation to effectiveness. A shorter time to obtain a volume of milk may be viewed as efficient for a woman pumping under time pressure. Simultaneous pumping protocols were shown to take less time to extract a volume of milk than sequential protocols; however, trials measure the actual time using the method in a controlled situation [18]. Mothers expressed views that simultaneous pumping of both breasts felt “cow-like”, awkward, did not allow a free hand, and they preferred sequential pumping even if slightly lower milk volume was obtained [26]. Trials did not report the time used for reading and understanding instructions, assembly, or cleaning of equipment, or the costs of double pump sets for simultaneous use—all aspects mentioned by mothers as important to them.

A high volume of milk may be viewed as effective for some situations. Use of techniques to assist milk flow, such as massage and relaxation, may assist in gaining higher milk volume in a shorter time [18]. Not all trials of pumps reported if any of these techniques were used by mothers in the trial.

Mothers participating in trials related to the establishment of breast milk supply in the period soon after birth may have high stress levels, fatigue, less privacy, and low confidence in their ability to produce adequate milk volumes, particularly if a first-time mother with an unexpected situation of her infant admitted to a neonatal unit. Trials may be easier for researchers to manage when the mothers are regularly at the neonatal unit; however, it is a challenge for studies to distinguish effectively between the overall experience of birth, commencing milk expression/pumping for a hospitalized infant, and the experience of using a specific device or method. Seven trials were carried out with mothers of healthy older infants at home, ranging from 3 to 35 weeks of age, who were feeding effectively at the breast and mothers were using the trial pumps specifically for trial purposes. The milk supply as well as the interest or motivation of mothers of older healthy infants to use a pump or pumping method are likely to be different from the mothers of newborn hospitalized infants establishing a milk supply in stressful conditions, who formed the sample in 11 trials. Readers of existing trials need to take into account the age and health of the baby and the stage of breastfeeding when evaluating trial findings.

The variability of the measurement instruments used presents a challenge to establishing the validity and reliability of the instruments and thus the comparability of the methods and devices. Most of the studies provided no information on the psychometric properties of their instruments. Lack of reliability and validity testing of instruments is noted in many areas of user satisfaction studies [24]. The majority of the instruments were developed and used in higher-income countries. The validity of breastfeeding-related instruments in cross-cultural studies has been raised [47,48]. Not all the trials reported any validity testing of the rating statements or validity testing when an instrument designed in one culture was used in another culture.

There was a noticeable chronological effect, with most of the pre-2001 trials gathering mothers’ views by means of simple questionnaires and reported descriptively; from 2001, rating scales became more common.

The design varied for the trials using rating scales (Table 3). In some trials, 1 was the least favourable end and, in others, 1 was the most favourable end, with one trial having a scale including zero. Some trials used descriptive anchors on the scale to aid a common understanding between respondent and researcher. These design variations impede comparability.

Scales with a five-point or seven-point scale permit a neutral mid-point and lower the usability of the responses. Does 4 on a scale of 7 indicate no strong view, undecided, or some other viewpoint? When the trial sample size is small, after seeing the scores, researchers may combine categories on the scale with few responses, which can have the effect of placing different emphases on the outer scores; for example, Meier 2008 collapsed five points into three categories, where a rating of 1–3 (out of 5) was analyzed as disagreement. How the results changed according to how the categories were collapsed was analyzed by one research team, finding a significant difference for some of the aspects measured depending on how mid-points were treated [27]. To address this issue of potential bias with Likert-type rating scales, this research team have changed to using a linear analogue scale so as to obtain results as a continuous variable and avoid the need for recoding [41].

The analysis methods used for rating scales also varied (Table 3). Some of the trials presented the number and percentage giving each rating, thus treating the data from the rating scale as discreet or ordinal data that indicated the relative position of responses but not the magnitude of difference. Other researchers treated the data from the rating scale as continuous data, with the assumption that the interval between a rating of 2 and of 3 (for example) signified the same magnitude of difference as the interval between 3 and 4, and presented results with a measure of central tendency (mean or median) and measures of spread (standard deviation). Researchers also used a binomial form by presenting results as the sum of positive and negative responses, or greater or less than a point. The statistical tests used varied in relation to how the scales were treated.

A mother’s rating and response to questions may be influenced by age or education, previous experience, expectations, motivation, gratitude to healthcare staff and any fear of reprisal for negative ratings, and co-interventions such as staff knowledge and support, staffing levels, mother’s access to her baby, rest, food, and fluids as well as incentives, such as being allowed to keep using a particular pump if she rates it higher [18,24,49]. Rating scales may tend towards more positive ratings and limited information, though these may be quicker to analyze. A combination of carefully designed rating scales and open questions may enable respondents to elaborate on their ratings and may provide a deeper understanding of their views [49,50].

Eight of the trials reviewed were cross-over trials designed to provide a comparison of two or more methods by the same study participant, thereby eliminating between-participant variation as well as enabling the participant to make a comparative judgement (Table 2). However, not all the cross-over studies reported the data with pair analysis or between-mother differences, thus negating the purpose of using a cross-over design [18].

Stating a preference between aspects of two or more pump devices or methods is different from measuring satisfaction [14]; it may be a situation of which is less bad rather than which is good. In trials comparing one device to another device, the women may be given a predetermined list of aspects of the pump to rate or comment on so as to focus the responses on the aspects deemed important to that trial. These aspects may be determined in relation to commercial needs for design testing or for marketing the device, such as rating the comfort of a newly designed pump compared to another pump. Though structured as a clinical trial of a medical device for assisting infants in receiving human milk, sometimes, results may be presented as a consumer marketing trial.

### Considerations for Future Research

This review highlights a need for standardization of core outcomes related to milk expression/pumping trials and thus the instruments used to measure how interventions may be achieving those outcomes. There is no agreed core outcomes set [51] for effectiveness research related to expressing and pumping breast milk. Current trial outcomes, which are published, may be those viewed as important by manufacturers in the marketing of their pump, with trial funding often linked to commercial interests [18]. Pump users may come to believe that the important aspects of a pump are those aspects most heavily marketed and this marketing may also influence researchers in their choice of outcomes to include [52].

Further work, including independent surveys, focus groups, and human-centered design approaches [11,13], would be helpful to establish which outcomes related to milk expression are relevant to mothers in various situations. There are clearly different motivations and needs in situations, such as a very preterm infant in a neonatal unit and a healthy older baby occasionally separated from their mother, or a low-resource setting.

Future research should mention how the pump users were involved in the research design and determination of the outcomes to be investigated [53]. The effect on the infant is also critical, particularly if the infant is fully dependent on pumped or expressed mother’s milk, when outcomes such as milk constituents become important, though mothers may be less aware of this aspect than health professionals caring for the infant [12]. The common survey finding of pain and discomfort highlights the need to have a better understanding of the concepts in relation to pumping—for example, is “not comfortable” similar to “painful”?—towards a common terminology. Other aspects relevant to mother’s viewpoint that could be considered for inclusion in future trials related to milk expression/pumping include cost and value for money, and environmental and sustainability components [18,54].

At the trial design stage, planning needs to minimize the possible effects of bias, confounding, and chance, including the timing with regard to establishment of milk supply, the situation and motivation for milk expression/pumping, co-interventions and support, and consideration that there may be changes in mothers’ views as they gain experience.

Instruments that are designed with attention to psychometric properties, contextual and cultural appropriateness, validity, and statistical robustness would aid in ensuring the completeness and reliability of results.

## 5. Conclusions

This review and secondary analysis examined which viewpoints of mothers were considered and how these were measured across 18 RCTs related to milk expression/pumping towards informing critical reading of research related to milk pumping as well as informing future research design.

Trials which sought mothers’ views on aspects of the expressing/pumping method did not appear to be examined or reported on with the same degree of rigor or attention as applied to other research outcomes, such as the quantity of milk obtained. There was wide variation in the aspects chosen by trialists, in the design of the research, in analysis used, and in how findings were reported. Work is needed to overcome the lack of standardized concepts and outcomes in this area of research.

Critical readers of existing research and new research undertaken on mother’s viewpoints are recommended to be aware and take account of bias that may occur either intentionally or unintentionally, through the design, analysis, and reporting of trials, and how this may influence the use of trial findings.

Key points when considering the mother’s viewpoint include what mothers are asked, how it is asked, and how the data collected are analyzed and reported. A clear methodologic base would improve the quality of this area of research.

Independent quality research would facilitate more children to receive human milk when direct breastfeeding is not feasible, thus having a positive influence on their health and wellbeing.

## Figures and Tables

**Table 3:** Fewtrell scale and adaptations.

Trial	Aspects to Rate
Fewtrell 2001b	Five: ease of use, amount of suction, comfort, pleasant to use, and overall opinion of the pump. Likert-type scale 1–7, 1 being most favorable, text anchors not reported.
Bernabe-Garcia 2012	Same as Fewtrell 2001b
Fewtrell 2001a	Same as Fewtrell 2001b
Hopkinson 2009	Seven: ease of use (very hard to very easy), comfort (very uncomfortable to very comfortable), strength of suction (too weak to too strong), feeling of suction (liked not at all to liked very much), sound (very annoying to not at all annoying), assembly (very hard to very easy), and overall (liked not at all to liked very much) Likert-type scale 1–7, 1 was least favorable.
Burton 2013	Nine: comfort, ease of assembly, ease of use, level of suction, noise level, flexibility regarding the rate and amount of suction, location of control button, speed of milk flow, and overall opinion of the pump. Likert-type scale 1–7, Reported as using 1 (very good) to 7 (very bad).
Fewtrell 2019	Nine: comfort, ease of use, how pleasant to use, suction, speed of milk flow, assembly, cleaning, leakage, and overall opinion are listed in the methods section of the published paper, which also reports results on “feel of the pump insert” and “the need to lean forward”. 10-cm visual analogue scale used with favorable on the left side.

## Data Availability

No datasets were generated during this review.
